# Metabolic responses to mild cold acclimation in type 2 diabetes patients

**DOI:** 10.1038/s41467-021-21813-0

**Published:** 2021-03-09

**Authors:** Carlijn M. E. Remie, Michiel P. B. Moonen, Kay H. M. Roumans, Emmani B. M. Nascimento, Anne Gemmink, Bas Havekes, Gert Schaart, Esther Kornips, Peter J. Joris, Vera B. Schrauwen-Hinderling, Joris Hoeks, Sander Kersten, Matthijs K. C. Hesselink, Esther Phielix, Wouter D. van Marken Lichtenbelt, Patrick Schrauwen

**Affiliations:** 1 Department of Nutrition and Movement Sciences, NUTRIM School for Nutrition and Translational Research in Metabolism, Maastricht University Medical Center, Maastricht, MD The Netherlands; 2 Department of Internal Medicine, Division of Endocrinology and Metabolic Disease, Maastricht University Medical Center, Maastricht, AZ The Netherlands; 3 Department of Radiology and Nuclear Medicine, Maastricht University Medical Center, Maastricht, AZ The Netherlands; 4 Nutrition, Metabolism and Genomics Group, Division of Human Nutrition and Health, Wageningen University, Wageningen, WE The Netherlands

**Keywords:** Diabetes, Endocrine system and metabolic diseases

## Abstract

Mild cold acclimation for 10 days has been previously shown to markedly improve insulin sensitivity in patients with type 2 diabetes. Here we show in a single-arm intervention study (Trialregister.nl ID: NL4469/NTR5711) in nine patients with type 2 diabetes that ten days of mild cold acclimation (16–17 °C) in which observable, overt shivering was prevented, does not result in improved insulin sensitivity, postprandial glucose and lipid metabolism or intrahepatic lipid content and only results in mild effects on overnight fasted fat oxidation, postprandial energy expenditure and aortic augmentation index. The lack of marked metabolic effects in this study is associated with a lack of self-reported shivering and a lack of upregulation of gene expression of muscle activation or muscle contraction pathways in skeletal muscle and suggests that some form of muscle contraction is needed for beneficial effects of mild cold acclimation.

## Abstract (web summary)

Cold acclimation has been shown to have beneficial metabolic effects, including improved insulin sensitivity in patients with type 2 diabetes. Here the authors show that a mild cold acclimation regiment during which overt shivering was prevented did not result in improved insulin sensitivity in a small group of patients with type 2 diabetes.

## Introduction

Mortality rates in type 2 diabetes mellitus (T2DM) patients are approximately twice as high compared to individuals without T2DM and can mostly be attributed to an increased risk of coronary heart diseases^1,2^. In the last decade, cold exposure as a tool to alleviate insulin resistance has attracted lots of scientific interest. Nowadays, there is little demand for the human body to adjust to colder temperatures, since humans nowadays spend most of their time in a well-controlled indoor environment with optimal temperatures within the body’s thermoneutral zone. The physiological reaction of the human body to cold exposure includes simultaneously (1) insulative responses by peripheral vasoconstriction and (2) an increase in metabolic rate by shivering thermogenesis (ST) and/or non-shivering thermogenesis (NST). It has been shown that during daily cold exposure in humans, shivering gradually decreases within 10–20 days, while the related increase in metabolic rate remains at a stable level^3–5^. This indicates that acclimation to cold occurs and that ST can be replaced by NST. Both brown adipose tissue (BAT)^6–9^ and skeletal muscle^4,10^ have been identified as contributors to NST.

Cold acclimation has been shown to promote insulin sensitivity in humans, originally attributed to increased BAT activity^11,12^. Research performed within our group, however, has shown that 10 days of cold acclimation (14–15 °C) markedly improved skeletal muscle insulin sensitivity by 43% in patients with type 2 diabetes^13^, an improvement that is comparable to the effect seen after long term exercise training^14^. Interestingly, the cold induced improvement of insulin sensitivity did not originate from BAT activation but was associated with increased GLUT4 translocation in skeletal muscle^13^.

Cold exposure may also impact other aspects of metabolic health, such as postprandial metabolism. Besides hyperglycemia, dyslipidemia is a common metabolic abnormality in patients with T2DM^15^. Interestingly, animal studies have shown reduced postprandial lipids upon prolonged cold exposure^16^. Moreover, a postprandial reduction in hypertriglyceridemia in human individuals was associated with BAT^17^. Lowering postprandial hyperglycemia and hypertriglyceridemia is clinically relevant as high plasma glucose and triglyceride levels can cause damage to the vascular wall inducing an impaired vascular function^18^, which causes atherosclerotic plaque development that may ultimately lead to cardiovascular disease events. However, the effect of cold exposure on these cardiovascular risk markers has not yet been investigated in humans.

Therefore, we primarily aimed to investigate the effect of 10 days of mild cold acclimation without overt shivering in overweight and obese patients with T2DM on postprandial glucose and lipid metabolism and cardiovascular risk markers. Second, we investigated whether a ten-day mild cold acclimation period affects insulin sensitivity, and if so, if this effect is sustained for another 10 days at room temperature. Given the previous results on skeletal muscle^13^, we also aimed to investigate if the effects of cold exposure can be achieved when skeletal muscle activity is prevented, and hence took specific care to prevent overt, observable shivering in our participants. We show no effects on postprandial glucose and lipid metabolism, and only mild effects on overnight fasted fat oxidation, postprandial energy expenditure, and aortic augmentation index. Furthermore, we show no effects on insulin sensitivity at both timepoints. No self-reported shivering during mild cold acclimation has been reported and we show no upregulation of gene expression of muscle activation or muscle contraction pathways in skeletal muscle after mild cold acclimation.

## Results

### Participant characteristics

**Fig. 1:**
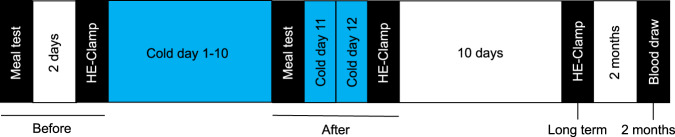
Study design. A meal test and hyperinsulinemic euglycemic clamp (HE-clamp) were performed before mild cold acclimation, separated by 2 days. After 10 days of cold exposure a second meal test was performed, followed by 2 additional days of cold exposure and followed by a second hyperinsulinemic euglycemic clamp. Ten days after the last intervention day, a third hyperinsulinemic euglycemic clamp was performed (long term). Two months after the last hyperinsulinemic euglycemic clamp another blood sample was collected. The blue boxes represent cold acclimation intervention (16–17 °C). The white boxes represent no intervention. The black boxes represent test days.

Nine obese men and women (age 65 ± 5 years; BMI 32.1 ± 2.8 kg m^−2^; four women) participated in the study (see Table 1, Fig. 1). In line with the inclusion criteria, participants were diagnosed for at least one year with type 2 diabetes, and were treated with oral medication only (see Supplementary Table 1). Participants were non-smokers, had no other active diseases, and had a sedentary lifestyle according to the Baecke questionnaire score (7.51 ± 1.16, Table 1).

**Table 1:** Participant characteristics.

Parameter	Mean ± SD
Gender F/M	4/5
Age (years)	65 ± 5
Body weight (kg)	93.7 ± 17.3
Height (m)	1.70 ± 0.10
BMI (kg m^−2^)	32.1 ± 2.8
HbA1c (%)	7.3 ± 0.7
TG (mmol l^−1^)	1.54 ± 0.34
ASAT (U L^−1^)	24 ± 8
ALAT (U L^−1^)	37 ± 19
GGT (U L^−1^)	34 ± 13
eGFR (ml min^−1^ 1.73 mm^−2^)	79 ± 9
Physical activity level (Baecke score)	7.51 ± 1.16

*F* female, *M* male, *BMI* body mass index, *HbA1c* hemoglobin A1c, *TG* triglycerides, *ASAT* aspartate aminotransferase, *ALAT* alanine aminotransferase, *GGT* gamma-glutamyl transferase, *eGFR* estimated glomerular filtration rate according CKD-EPI method.

### Body and room temperature, thermal comfort, and shivering during cold acclimation

Room temperature during cold acclimation was on average 16.4 ± 0.30 **°**C, which was ~1.4 **°**C higher compared to the previous study by Hanssen et al.^13^. Average skin temperature dropped from 27.5 ± 0.33 **°**C to 26.3 ± 0.97 **°**C during day 3. This was similar to the drop in average skin temperature from 27.4 ± 0.80 **°**C to 26.4 ± 1.26 **°**C on day 10. Thermal sensation and thermal comfort, assessed via VAS scales, were not significantly different between day 3 and day 10 of cold acclimation, as shown in Supplementary Fig. 1. During the cold acclimation sessions on day 3 and 10, the temperature was progressively perceived colder and more uncomfortable over time. The shivering questionnaires revealed that participants experienced no shivering and only occasionally reported tense muscles. Self-reported shivering intensity was less than described in the study from Hanssen et al.^13^ and were not significant different between day 3 and day 10 of cold acclimation.

### Postprandial metabolism and substrate kinetics

**Fig. 2:**
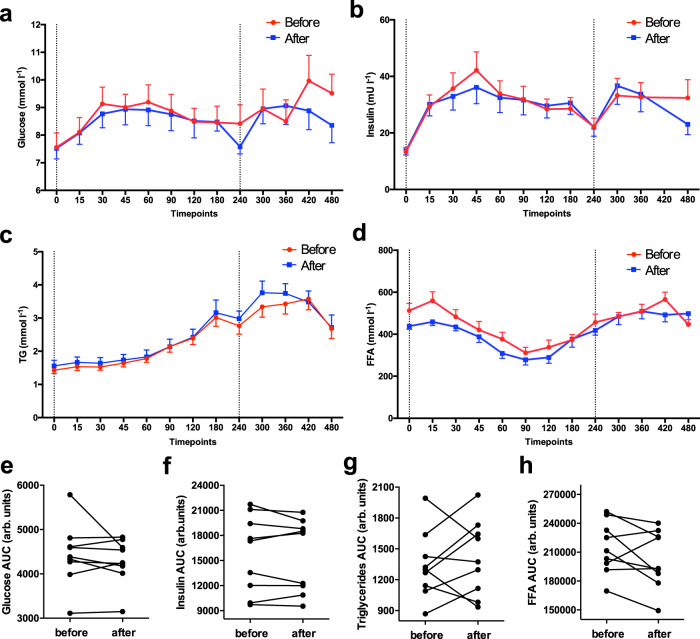
Plasma levels during the meal tests. **a** Plasma levels of glucose (*n* = 9). **b** Insulin (*n* = 9). **c** Triglycerides (*n* = 9). **d** Free fatty acids during the meal test (*n* = 9). **e**–**h** Corresponding area under the curves (AUC) (*n* = 9). Before mild cold acclimation is presented as the red line, after mild cold acclimation as the blue line. Dashed vertical lines indicate the time of consumption of the 1st shake at T0 and the 2nd shake at T240. Data was analyzed with a two-sided Wilcoxon matched-pairs signed rank test. No significant differences were observed (all *p* > 0.05). Data is presented as mean ± SE and individual datapoints.

Meal test total area-under-the-curve values (AUC) for glucose, insulin, and triglycerides were not significantly different before and after cold acclimation (*p* = 0.43, *p* = 0.65, and *p* = 0.50, respectively, Fig. 2a–c and Supplementary Table 4). In addition, no significant differences were observed in AUC when the 1st or 2nd meal of the meal test were analysed separately (Supplementary Table 4). Area under the curve for plasma free fatty acids was also not significantly different before and after 10 days of cold acclimation (*p* = 0.16, Fig. 2d and Supplementary Table 4). However, when the 1st and 2nd meal of the meal test were analysed separately, the total AUC for FFA during the 1st meal was significantly lower after cold acclimation (before: 94228 ± 5115 mmol/l; after: 86014 ± 4545 mmol/l, *p* = 0.039) as shown in Supplementary Table 4.

**Fig. 3:**
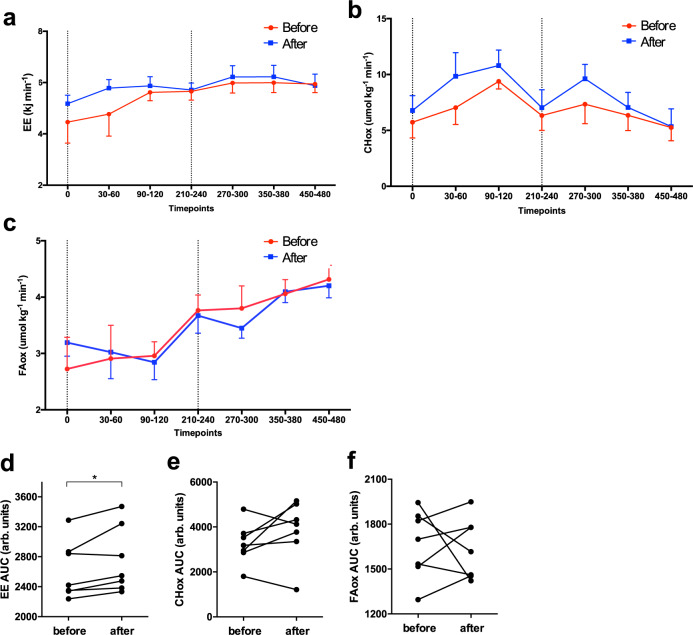
Energy expenditure and substrate oxidation during the meal tests. **a** Energy expenditure (*n* = 7). **b** Carbohydrate oxidation (CHox) (*n* = 7). **c** Fatty acid oxidation (FAox) (*n* = 7) during the meal tests. **d**–**f** Corresponding area under the curves (AUC) (*n* = 7), EE AUC *p* = 0.03. Before mild cold acclimation is presented as the red line, after mild cold acclimation as the blue line. Dashed vertical lines indicate the time of consumption of the 1st shake at T0 and the 2nd shake at T240. Data was analyzed with a two-sided Wilcoxon matched-pairs signed rank test. Data is presented as mean ± SE and individual datapoints. *p < 0.05.

Total energy expenditure during the meal tests, calculated as AUC, was significantly higher after the cold acclimation (before: 2620 ± 146; after: 2752 ± 168 kJ, *p* = 0.03, Fig. 3a, d and Supplementary Table 4). Although this elevated energy expenditure seemed to be mainly due to higher glucose oxidation, the differences in glucose and fat oxidation during the meal tests were not statistical significant (*p* = 0.44 and *p* > 0.99, respectively, Fig. 3b, c, Supplementary Table 4).

The results of the meal test suggest that cold acclimation may increase energy expenditure, probably due to higher carbohydrate oxidation. To further test this, we analysed substrate oxidation measured in the morning of the hyperinsulinemic euglycemic clamp. Overnight fasted energy expenditure at the start of the hyperinsulinemic euglycemic clamp (baseline) was not affected by cold acclimation (Supplementary Table 3). However, energy expenditure during the high insulin phase was significantly higher after cold acclimation compared to before (before 4.67 ± 0.30 vs. after 4.89 ± 0.27 kJ min^−1^, *p* = 0.03) and this elevation tended to return to baseline levels on the long term (after 4.89 ± 0.27 vs. long term 4.67 ± 0.18 kJ min^−1^, *p* = 0.10, Supplementary Table 3). Carbohydrate oxidation after an overnight fast was significantly higher after 10 days of cold acclimation compared to before (before 3.76 ± 0.47 vs. after 4.97 ± 0.68 μmol kg^−1^ min^−1^, *p* < 0.01, Supplementary Table 3). In addition, fat oxidation after an overnight fast was significantly lower directly after cold acclimation compared to before (before 3.80 ± 0.17 vs. after 3.57 ± 0.17 μmol kg^−1^ min^−1^, *p* < 0.01, Supplementary Table 3). These effects on carbohydrate and fat oxidation did not sustain on the long term (Supplementary Table 3). The changes in substrate oxidation after cold acclimation were not observed during insulin infusion (Supplementary Table 3).

There was no significant difference after cold acclimation for baseline HDL (before 1.12 ± 0.12 vs. after 1.21 ± 0.08 mmol l^−1^, *p* = 0.16), LDL (before 1.80 ± 0.24 vs. after 1.84 ± 0.24 mmol l^−1^, *p* = 0.82), or total cholesterol (before 3.57 ± 0.31 vs. after 3.76 ± 0.31 mmol l^−1^, *p* = 0.16).

### Vascular function

**Fig. 4:**
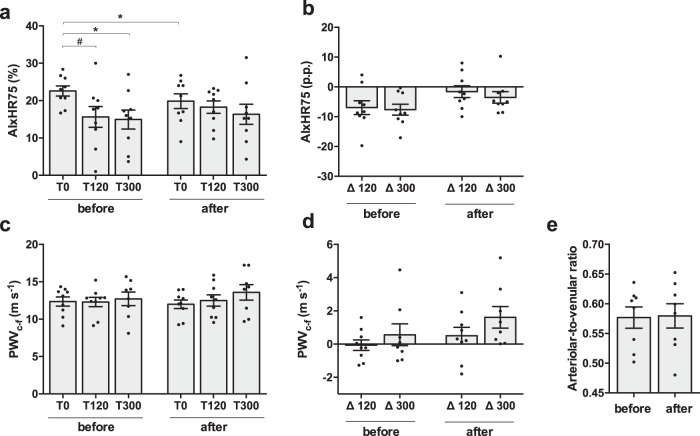
Vascular function markers. **a** Aortic augmentation index (AIxHR75) at timepoints T0, T120, T300 (*n* = 9), before T0 vs. after T0 *p* = 0.03, before T0 vs. before T120 *p* = 0.10, before T0 vs. before T300 *p* = 0.02. **b** AIxHR75 delta’s T0-120 and T0-300 expressed as percentage points (*n* = 9). **c** Pulse wave velocity (PWV_c-f_) at timepoints T0, T120, T300 (*n* = 8 at T300). **d** PWV_c-f_ delta’s T0-120 and T0-300 (*n* = 8 at delta T0-300). **e** Arteriolar-to-venular ratio of the retinal vessels in the right eye (*n* = 8). Data was analyzed with a two-sided Wilcoxon matched-pairs signed rank test. Data shown are shown as individual datapoints and mean ± SE. *p < 0.05, ^#^*p* < 0.10.

AIxHR75, an indirect marker of arterial stiffness, was significantly improved after 10 days of cold acclimation measured in the overnight fasted state (before T0 22.57 ± 1.36% vs. after T0 19.84 ± 1.96%, *p* = 0.03, Fig. 4a). Before cold acclimation, AIxHR75 tended to decrease upon the first meal ingestion (T120) and this meal-induced effect became significant after the second meal (T300). No statistically significant meal-effects on AIxHR75 were observed after cold acclimation (before T120 15.63 ± 2.80% vs. after T120 18.26 ± 1.67% *p* = 0.50 and before T300 14.94 ± 2.55% vs. after T300 16.33 ± 2.70% *p* = 0.82, Fig. 4a). Postprandial changes in AIxHR75 were also not significant different before and after cold acclimation (before delta 120 −6.94 ± 2.31 vs. after delta 120 −1.58 ± 1.96, *p* = 0.16, before delta 300 −7.63 ± 1.83 vs. after delta 300 −3.51 ± 1.91, *p* = 0.30, Fig. 4b). The current non-invasive gold standard technique to measure arterial stiffness (PWV_c-f_), was however not affected by cold acclimation in the overnight fasted state PWV_c-f_ (before T0 12.36 ± 0.61 vs. after T0 11.99 ± 0.57, *p* = 0.36, Fig. 4c). As expected, no meal-induced effects were observed (see Fig. 4d).

Finally, 10 days of mild cold acclimation did not significantly affect fasting retinal vessel diameter, as the arteriolar width (before 120.83 ± 7.48 μm vs. after 120.52 ± 7.89 μm, *p* = 0.95), venular width (before 209.19 ± 10.73 μm vs. after 207.17 ± 10.07 μm, *p* = 0.23) and the arteriolar-to-venular ratio (before 0.58 ± 0.02 vs. after 0.58 ± 0.02, *p* = 0.84, Fig. 4e) did not change.

### Liver fat content

**Fig. 5:**
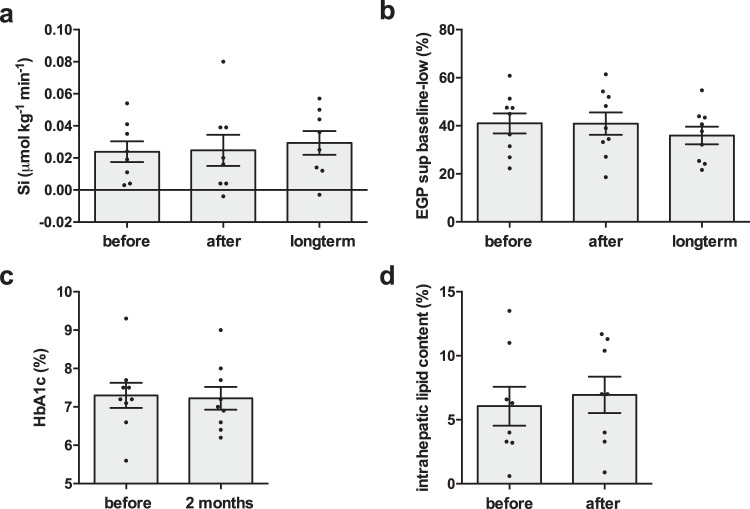
Insulin sensitivity and intrahepatic lipid content. **a** Whole body insulin sensitivity (*n* = 8). **b** Suppression of hepatic endogenous glucose production (*n* = 9). **c** HbA1c before the cold acclimation and 2 months after the cold acclimation (*n* = 9). **d** Intrahepatic lipid content (*n* = 8). Data was analyzed with a two-sided Friedman test for Si and EGP. Data was analyzed with a two-sided Wilcoxon matched-pairs signed rank test for HbA1c and intrahepatic lipid content. Data are shown as individual datapoints and mean ± SE.

As we hypothesized that mild cold acclimation could affect postprandial lipid metabolism, we also investigated if mild cold acclimation affects liver fat content. However, liver fat content was not different before and directly after mild cold acclimation (before 6.1 ± 4.3 vs. after 7.0 ± 4.0%, *p* = 0.22, *n* = 8, Fig. 5d).

### Insulin sensitivity

We previously demonstrated that mild cold acclimation improved insulin sensitivity when measured directly after 10 days of cold acclimation. Here, we aimed to investigate if this increase in insulin sensitivity would be sustained for 10 days after cessation of the cold acclimation intervention. Plasma insulin levels during the high insulin phase of the hyperinsulinemic euglycemic clamp were significantly different between test days (*p* = 0.02) with significantly higher values directly after the cold acclimation compared with before cold acclimation (*p* = 0.02, Supplementary Table 3), and therefore we calculated Si as a measure of insulin sensitivity. However, whole body insulin stimulated glucose uptake (Rd glucose high insulin minus baseline) corrected for plasma insulin levels (Si), was not different before and directly after cold acclimation nor on the long term (*p* = 0.53, Fig. 5a). Hepatic insulin sensitivity during the low insulin phase was also not affected by cold acclimation (*p* = 0.28, Fig. 5b), however during the high insulin phase, EGP suppression was significantly lower directly after cold acclimation compared with before and on the long term (before 88.06 ± 7.00% vs. after 77.60 ± 7.45% vs. long term 88.39 ± 3.44%, *p* = 0.04). HbA1c, a marker of long term glucose homeostasis, was not different before cold acclimation compared with 2 months after cold acclimation (before 7.3 ± 0.3% vs. 2 months later 7.2 ± 0.3%, *p* = 0.71, Fig. 5c).

### Skeletal muscle GLUT4 translocation

**Fig. 6:**
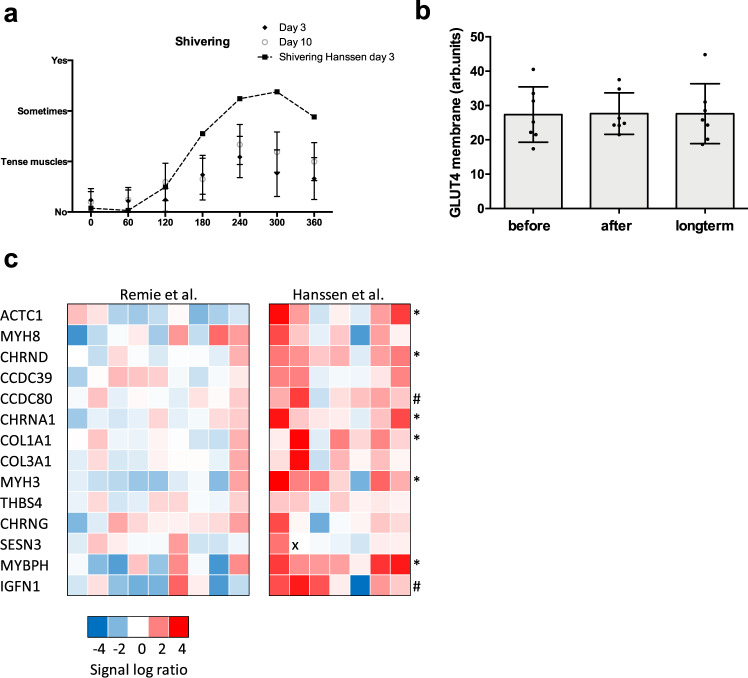
Skeletal muscle activity markers. **a** Self-reported shivering measured with VAS scales at selected timepoints (from T = 0 min until T = 360 min) during day 3 (*n* = 9) and day 10 (*n* = 9) of the mild cold acclimation period, shown as AUC. The dashed line indicates the self-reported shivering AUC from Hanssen et al.^13^ at day 3 (*n* = 8). **b** GLUT4 intensity at the skeletal muscle membrane, expressed as arbitrary units (arb.units) (*n* = 7). **c** Relative gene expression analysis in skeletal muscle biopsy samples obtained from the current study (Remie et al.) (*n* = 9) and from Hanssen et al.^13^ (*n* = 7) before and after mild cold acclimation. qPCR data is expressed as heatmap. Amplification failed for one sample and has been identified by “x” in the heat map. Data was analyzed with a two-sided Wilcoxon matched-pairs signed rank test for shivering. Data was analyzed with a two-sided Friedman test for GLUT4. Data was analyzed with a two-sided Mann–Whitney test for relative gene expression. Data are shown as mean ± SE. *p < 0.05, ^#^*p* < 0.10.

The lack of effect of mild cold acclimation without observable, overt shiving on insulin sensitivity contrasts our previous study, in which we found that the increased insulin sensitivity upon mild cold acclimation was due to enhanced GLUT4 translocation in skeletal muscle^13^. Consistent with a lack of effect of mild cold acclimation on insulin sensitivity in the current study, GLUT4 intensity at the membrane measured in muscle biopsies taken in the non-insulin stimulated condition, was also not affected by mild cold acclimation (before 27.37 ± 3.05 vs. after 27.63 ± 2.28 vs. long term 27.61 ± 3.30 arb. units, *p* = 0.77, *n* = 7, Fig. 6b). Cytosolic GLUT4 intensity was not changed either (before 20.21 ± 1.89 vs. after 20.71 ± 1.33 vs. long term 20.46 ± 1.90 arb. units, *p* = 0.96).

### Skeletal muscle shivering markers

Because we could not replicate our previously reported positive effects of mild cold acclimation on insulin sensitivity and GLUT4 translocation^13^, we decided to investigate if the absence of overt, observable shivering in the current study could underly this phenomenon. Compared with our previous study^13^, we see a distinct lack of self-reported shivering, as can be seen in Fig. 6A. Since we did not perform electromyography (EMG), neither in the previous nor the current study, we next investigated if a certain degree of skeletal muscle activation may underly the beneficial effects on insulin sensitivity observed in our previous study. To this end, we measured the transcripts of several genes that were selected based on previously performed microarray analyses in skeletal muscle derived from our previous cold acclimation study^13^ and an exercise intervention study^14^. Based on these array data, we selected genes related to muscle contraction and the extracellular matrix^19^. Based on the heatmap, showing the expression of the selected genes in the previous and the current mild cold acclimation study, a clear upregulation in these genes could be observed in the original cold acclimation study from Hanssen et al.^13^, whereas this expression pattern was completely absent in the current study (Fig. 6c). In more detail, smooth muscle actin (ACTC1), the alpha and delta subunit of muscle acetylcholine receptor (CHRNA1, CHRND), alpha-1 type-1 collagen (COL1A1), skeletal muscle myosin heavy chain 3 (MYH3), and myosin binding protein H (MYBPH) were higher in the cold acclimation study of Hanssen et al.^13^ compared with our current study. Also coiled coil domain containing protein 80 (CCDC80) and immunogloblulin-like and fibronectin type III domains-containing protein 1 (IGFN1) showed tendancies to be increased in the cold acclimation study from Hanssen et al.^13^ compared with our current study.

## Discussion

Previous studies investigating cold acclimation in humans have shown a potential to treat obesity and T2DM via an increase in energy expenditure^4–8,12,13,20,21^ and insulin sensitivity^13^, acting through brown adipose tissue and skeletal muscle. Therefore we hypothesized that mild cold acclimation could also be beneficial for postprandial metabolism and reduce cardiovascular risk. To this end, we primarily investigated the effects of ten days of mild cold acclimation on cardiovascular risk markers, including postprandial glucose and lipid metabolism and markers for arterial stiffness. Moreover, we investigated the long term effects of mild cold acclimation on insulin sensitivity.

Acute cold exposure with and without shivering has frequently been shown to increase energy expenditure, reported as an increase in basal metabolic rate^4,5,10,11,17,20–24^. Yet, consistent with other previous reports^4,6–8,12,13,21,25^, we did not observe an increase in basal metabolic rate, measured under thermoneutral conditions after mild cold acclimation without observable, overt shivering. However, our results show a change in fasting substrate selection after mild cold acclimation, with a decrease in fat oxidation and an increase in carbohydrate oxidation. Furthermore, as also reported before^12^, postprandial energy expenditure that reflects diet induced thermogenesis, was higher in the meal test after cold acclimation compared to the meal test prior cold acclimation. Other evidence suggests that acute cold exposure^5,11,16,17,22^, but not cold acclimation^5^, increases glucose oxidation, fat oxidation, and lipid clearance. In agreement with previous work^5,24^, we did not observe changes in fasting plasma lipid levels, postprandial substrate oxidation, or postprandial triglyceride response. We only observed a small, albeit significant decrease in plasma fatty acid levels during the first step of the meal test after cold acclimation. Furthermore, no effects of mild cold acclimation were observed on fasted plasma glucose and insulin levels, consistent with findings after acute cold exposure^24^. These results indicate that mild cold acclimation under conditions where overt shivering is prevented only has marginal effects on postprandial glucose and lipid metabolism.

Furthermore, we observed a significant improvement in the aortic augmentation index, but PWV_c-f_ did not change. This indicates that ten-day mild cold acclimation already affects the resistance (tone) of the arteries that is reflected by a reduced AIxHR75. However, our study period was most likely too short to affect arterial stiffness by altering structural properties of arterial walls, as measured by PWV_c-f_^26^.

We previously showed marked effects of cold acclimation on insulin sensitivity in T2DM patients^13^. Here we aimed to investigate if these effects would be retained for a longer period of time after the last cold exposure. Thus, we performed a hyperinsulinemic euglycemic clamp before cold acclimation, directly (1 day) after cold acclimation and 10 days after the last cold exposure. Surprisingly, we could not replicate the increase in insulin sensitivity following the ten-day cold acclimation period, and also no effects on the long term were observed. Since the increase in insulin sensitivity in our previous study^13^ was very marked (40% increase), observed in all participants, and was accompanied by marked increases in muscle GLUT4 translocation, we carefully evaluated the differences between the two studies. Thus, in both studies patients with T2DM were investigated before and after ten days of cold exposure, following a similar design. One difference between both studies was the gender of the study participants as the current study included both men and women, whereas the previous study only included men. In addition, although inclusion criteria for age and body weight were similar in both studies, T2DM patients in the current study were on average slightly older (~65 vs. 60 years) and slightly heavier (94 vs. 92 kg) in comparison with the previous study. However, it should be noted that in our previous study, the increase in insulin sensitivity was very marked and observed in all participants, whereas in the current study insulin sensitivity was very slightly increased in 4 participants and decreased in 5 participants, with no differences in gender distribution. Therefore, we believe that the lack of effect of mild cold acclimation in the current study cannot be explained by gender or small differences in participant characteristics. Another difference in the current study design was the inclusion of a meal test as the primary outcome parameter. This meal test was performed on the day following the ten day cold exposure. Consequently, the hyperinsulinemic euglycemic clamp in the current study was performed three days after the meal test, with two days of cold exposure in between. We can hence not exclude a carry-over effect of the meal test on the results of the hyperinsulinemic euglycemic clamp. It should be noted though, that postprandial glucose metabolism measured during the meal tests that can be seen as a marker of insulin sensitivity, was also not affected by cold acclimation. Another difference between the two studies was that in the current study we specifically aimed to prevent shivering. To this end, the room temperature during cold exposure was ~1.4 **°**C higher compared with our previous study^13^. Furthermore, participants were provided with extra clothing when self-reported or observable shivering occurred to try to prevent shivering thermogenesis. Although we did not perform EMG in the current study, and therefore cannot exclude shivering thermogenesis, our strategy was at least effective in preventing self-reported shivering and/or feelings of tense muscle in comparison with our previous study^13^, as indicated by the self-reported shivering questionnaires taken during the cold acclimation. We previously reported that the increase in insulin sensitivity upon mild cold acclimation was paralleled by a marked increase in GLUT4 translocation in the overnight fasted state (so in the absence of elevated insulin levels)^7,13^. Classically, this increase in GLUT4 in the celmembrane in the absence of insulin stimulation is attributed to muscle contraction, which is required for shivering or to increase muscle tension^27^. Consistent with the lack of effect of mild cold acclimation on insulin sensitivity, no effect of cold acclimation on GLUT4 translocation was found in the current study. To further investigate the possible effects of shivering and skeletal muscle activation that we may have missed in our previous study^13^, we performed gene expression analyses in skeletal muscle biopsies obtained before and directly after the cold acclimation in both studies. The genes we measured were selected from a comparison of micro-array data obtained in skeletal muscle biopsies taken after mild cold exposure^13^ and after an exercise training study^14^. The results of that comparative transcriptome analysis revealed that cold acclimation and exercise training have overlapping effects on gene expression in human skeletal muscle, but strikingly these overlapping genes are designated to pathways related to skeletal muscle contraction pathways rather than metabolic pathways^19^. Interestingly, when we measured the mRNA expression of these selected genes in the current study, we observed no effect of mild cold acclimation, in contrast to our previous study^13^. Interestingly, all previous published studies that observed improvements in insulin sensitivity after cold acclimation, included at least a few days where (mild) shivering occurred^4,5,7,13,21^. Taken together, the findings reported here and in literature^4,10,28^, suggest that some mild form of muscle contraction or muscle activation during cold acclimation is needed to trigger beneficial effects on skeletal muscle insulin sensitivity.

In conclusion, we here show that a ten-day cold acclimation period at 16–17 °C does not induce metabolic improvements nor reduce cardiovascular risk markers in obese men and women with T2DM. The lack of effects in the current study are probably due to the absence of a sufficient level of skeletal muscle activation during cold exposure compared with previous studies. Therefore, further research could focus on the minimum level of skeletal muscle activation during cold exposure that is needed to translate into beneficial metabolic health effects after mild cold acclimation. In addition, our results may also hint towards the potential of (mild) shivering thermogenesis on metabolic health, but future studies are needed to investigate if repeated bouts of shivering may lead to long term metabolic health effects.

## Methods

### Participants

Ten obese men and women were included in the study with one participant dropping out because of personal reasons. Hence, nine participants completed the study. All participants underwent a screening including assessment of blood parameters, electrocardiography, anthropometric measurements, and a questionnaire to evaluate eligibility. Inclusion criteria were: 45–70 years of age, BMI 27–35 kg m^−2^, diagnosed with type 2 diabetes for at least 1 year (relatively well-controlled HbA1c <8.5%), use of oral glucose lowering drugs (metformin and/or sulfonylurea agents), a sedentary lifestyle (<3 h exercise per week), non-smoking for at least six months, no alcohol use of >2 servings per day and a stable body weight for at least 6 months. Other medication without known effects on the primary outcome measurement was allowed. Data were collected between March 15, 2016 and August 24, 2017.

### Ethical approval

The study was conducted according to the declaration of Helsinki and was approved by the Ethics Committee of the Maastricht University Medical Center. The study was registered at https://trialregister.nl (NL4469/NTR5711). All participants provided written informed consent before screening.

### Study design

Figure 1 provides an overview of the study design. A ten-day cold acclimation intervention was performed in which subjects were exposed to an environmental temperature of 16–17 °C to avoid overt shivering for ten consecutive days: 2 h on day one, 4 h on day two, and 6 h on days 3 through 10. Before the onset of the cold acclimation period a high-fat meal test (meal test before) was combined with several vascular function measurements. Three days later, liver fat content was measured followed by a muscle biopsy and a hypersinsulinemic-euglycemic 2-step clamp (HE- clamp before). Subsequently, a ten-day cold acclimation intervention started (Day 1–10). One day after the last cold exposure, (Day 11), the first test day was repeated (meal test after). The following 2 days (Day 12–13), additional cold exposure was maintained to ensure sustainment of the intervention effect for the re-tests on Day 14 (HE-clamp after). Upon ten days without intervention, another muscle biopsy was taken followed by a hypersinsulinemic-euglycemic 2-step clamp (HE-clamp long term) to assess the sustained long term effect of cold acclimation on whole body insulin sensitivity. Two months after the last test day, fasting blood samples were taken to measure markers for glucose homeostasis. All tests were executed at room temperature. At the evening preceding all test days, participants consumed a standardized meal and remained fasted from 20:00 h onwards. In addition, participants were asked to refrain for at least 48 h from any physical activity different from their daily routine. Participants arrived at the testing facility by car. Blood glucose lowering medication was continued during the entire study, except for the morning of the hyperinsulinemic euglycemic clamp.

### Cold acclimation

During cold acclimation, participants were progressively exposed to an environmental temperature of 16–17 °C for ten consecutive days: 2-h on day 1, 4-h on day 2 and 6-h on days 3–10. On Day 11–12, participants were again cold-exposed for 6 h. Participants were dressed in short-sleeved T-shirts and shorts and remained sedentary while staying in the cold room and instructed not to change their habitual diet during the entire study period. Food intake while staying in the cold room was kept constant. Wireless temperature sensors (iButtons, Maxim Integrated, San Jose, CA, USA) were placed on 15 ISO-defined sites on days 3 and 10 of the cold acclimation period to measure skin temperature. Participants were not allowed to shiver. In case observable shivering started, extra clothing was immediately provided, which the participant wore until shivering stopped. At selected timepoints during day 3 and 10 VAS scales on thermal sensation, thermal comfort and self-reported shivering were completed. Incremental AUCs (iAUC) were calculated to determine subjective responses during the cold acclimation period.

### High-fat meal test

After placing an intravenous cannula, a fasted blood sample was drawn (T = 0) and energy expenditure and substrate oxidation was measured via indirect calorimetry. At 09:00 h, participants were asked to consume a high-fat shake, within 10 min. The nutritional content of the shake is shown in Supplementary Table 2 and was similar for all participants. Subsequently, blood samples were drawn after 15, 30, 45, 60, 90, 120, 180, 240, 300, 360, 420, and 480 min. After the blood sampling at T = 240, at 13:00 h, the participants consumed a second high-fat shake with the exact same contents as in the morning to investigate the second-meal effect. At T30–60, T90–120, T210–240, T270–300, T350–380, and T450–480 indirect calorimetry was performed to measure energy expenditure and substrate oxidation. Participants were not allowed to eat or drink anything else throughout the test day, except for water.

### Vascular function measurements

In the morning of the meal tests, radial artery pulse wave analysis (PWA) was performed with a tonometer (SphygmoCor v9, AtCor Medical, West Ryde, Australia), from which the aortic augmentation index corrected for heart rate (AIxHR75) was calculated as the difference between the first peak and second peak of the arterial waveform, expressed as a percentage of the pulse pressure and corrected for heart rate. Using the same tonometer, carotid-to-femoral pulse wave velocity (PWV_c-f_) was determined by measuring the arrival of the pulse wave and the delay to the R-wave of the electrocardiogram at the carotid and femoral artery. PWVc-f was calculated automatically by the program of the manufacturer after entering 80% of the direct straight carotid-to-femoral distance. The vascular stiffness measurements were performed before (T0) and during the high-fat meal tests at two different time points (T120 and T300).

Retinal vascular images were obtained to measure microvascular effects. Retinal images were obtained using a nonmydriatic retinal camera (Topcon TRC-NW-300; Topcon Co.)^29^. At least two arteriolar and two venular segments were measured and summarized by using the Parr–Hubbard formulas^30^. These segments differed between study participants, however, they had to be exactly the same segments for each participant at all measurements.

### Hyperinsulinemic euglycemic clamp

To determine insulin sensitivity, a two-step hyperinsulinemic euglycemic clamp^31^ with co-infusion of D-[6.6-^2^H_2_] glucose tracer (0.04 mg kg^−1^ min^−1^) was performed. Insulin-suppressed endogenous glucose production (EGP) during low insulin infusion (10 mU  m^−2^ min^−1^) was measured as a reflection of hepatic insulin sensitivity. This was followed by a high insulin infusion (40 mU m^−2^ min^−1^) to measure whole body glucose disposal (Rd). Indirect calorimetry was performed during baseline, low insulin, and high insulin to measure energy expenditure and substrate oxidation. Steele’s single pool non-steady state equations were used to calculate glucose appearance (Ra) and glucose disposal (Rd)^32^. Volume of distribution was assumed to be 0.160 l kg^−1^ for glucose. Whole-body insulin sensitivity (S_i_) was calculated according to Bergman et al.^33^, taking differences in insulin and glucose levels into account: S_i_ = ΔRd/(Δinsulin*clamping glucose), where Δ represents the change from the basal state to the insulin-stimulated condition. EGP was calculated as Ra minus exogenous glucose infusion rate. Non-oxidative glucose disposal (NOGD) was calculated as Rd minus carbohydrate oxidation.

### Indirect calorimetry

Whole body oxygen consumption and carbon dioxide production were measured during specific timepoints in the meal test and the hyperinsulinemic euglycemic clamp using an automated respiratory gas analyzer with a ventilated hood system (Omnical; Maastricht Instruments, Maastricht, The Netherlands). Participants were measured in supine position for 30 min each time. Energy expenditure, glucose oxidation, and fat oxidation rates were calculated using equations based on the measured averaged oxygen and carbon dioxide concentrations with the assumption that protein oxidation was negligible^34,35^.

### Intrahepatic lipid quantification by MR spectroscopy

Proton magnetic resonance spectroscopy (^1^H-MRS) was used to quantify intrahepatic lipid content (IHL) at 07:00 h in the morning of the hyperinsulinemic euglycemic clamp before and after cold acclimation. All measurements were performed on a 3.0 T whole body scanner (Achieva Tx, Philips Healthcare, Best, The Netherlands). Liver spectra were obtained with the subject in supine position, head first. Images and MR spectra were obtained using a 32-element sense cardiac/torso coil (Philips Healthcare, Best, The Netherlands). ^1^H MR spectra were obtained from a 40 × 40 × 40 mm^3^ voxel, placed centrally in the right lobe of the liver, using point resolved spectroscopy volume selection with repetition time (TR) 4000 ms, echo time (TE) 32.5 ms and 2048 sample points. Values are given as T2 corrected ratios of the CH_2_ peak relative to the unsuppressed water peak, expressed as percentage.

### Skeletal muscle biopsies

On each hyperinsulinemic euglycemic clamp day, a muscle biopsy was taken from the *m. vastus lateralis* under local anesthesia (2% lidocaine, without epinephrine) using the Bergström technique^36^. The muscle biopsy was taken before the start of the insulin infusion on the morning of the hyperinsulinemic euglycemic clamp, at 08:30 h after an overnight fast. The biopsy was divided in several parts. One part was immediately frozen in melting isopentane for biochemical analyses. Other parts were embedded in Tissue-Tek and frozen in melting isopentane for immunohistochemical analyses.

### Histochemical analysis of GLUT4 in skeletal muscle biopsies

Muscle biopsies taken in the overnight fasted state, prior to all three hyperinsulinemic euglycemic clamps, were analysed for GLUT4 translocation. For GLUT4 imaging, double immunofluorescence assays were performed on 5 µm-thick fresh frozen tissue sections, which were fixated during 15 min with 3.7% formaldehyde in PBS and thereafter treated for 5 min with 0.5% Triton X-100 in PBS. Sections were incubated overnight at 4 °C with a mix of primary antibodies directed to GLUT4 (Santa Cruz, BioConnect, Huissen, The Netherlands) and laminin (Sigma, Zwijndrecht, The Netherlands). After three washing steps with PBS, AlexaFluor555, and AlexaFluor488 conjugated secondary antibodies were incubated for 45 min at room temperature. After a final washing step with PBS sections were mounted in Mowiol. Images were observed using a Nikon E800 fluorescence microscope with NIS-elements Imaging Software (Nikon Europe BV, Amsterdam, The Netherlands) and were captured with identical exposure time and gain settings in before- and after-conditions. Without any adjustments with respect to color intensity, brightness or contrast, RGB stacked images were quantified using the Plot Profile tool in Image J^37^. Thus, we measured the intensity of GLUT4 raised signals (16 bits) throughout the sections. Measured data on intensity were used to generate overlying plots of GLUT4 and laminin. The GLUT4 peak value in the membrane was divided over the mean GLUT4 content in 10 pixels located in the cytosol of the very same cell. Thus, a score >1.0 implicates that relatively more GLUT4 is detected in the membrane than in cytosolic regions and hence reflects GLUT4 translocation.

### Muscle activation markers in skeletal muscle biopsies

RNA was isolated from skeletal muscle biopsies using Trizol followed by purification using the RNeasy kit from Qiagen (Hildenberg, Germany). cDNA was created by using the high-capacity RNA-to-cDNA kit from Applied Biosystems (Foster City, USA). mRNA expression was determined using a CFX384 Touch Real-Time PCR Detection System from BioRad Laboratories (Hercules, CA). Gene expression was normalized to RPLPO and expression was analyzed via the standard curve method. Data is expressed in heatmap showing changes over basal using signal log ratios. Primer sequences and/or Taqman ID’s are listed in Supplementary Table 5.

### Blood sampling and analyses

Blood collected in EDTA-coated tubes were immediately stored on ice, centrifuged and plasma was stored at −80 °C until analyses. Blood collected in serum-tubes was stored at room temperature for at least 30 min to allow coagulation, followed by centrifugation and storage at −80 °C until analyses. Glucose (Hk-CP, Axonlab, Amsterdam, The Netherlands) and FFA (NEFA-HR, WAKO chemicals, Neuss, Germany) in meal test samples were analyzed enzymatically in EDTA plasma using a Pentra 400 (Horiba, Montpellier, France). Insulin during the meal test was analyzed using RIA, and insulin during the hyperinsulinemic euglycemic clamp was analyzed using ELISA. Triglycerides (Sigma, Zwijndrecht, The Netherlands), cholesterol (CHOD-PAP, Roche Diagnostics, Mannheim, Germany), and HDL-cholesterol (CHOD-PAP, Roche Diagnostics, Mannheim,Germany) after precipitation of apoB-containing lipoproteins with phosphotungstic acid and magnesium ions, were analyzed in serum also using a Pentra 400. LDL-cholesterol was calculated according the Friedewald equation^38^.

### Statistical analyses

Participant characteristics are reported as mean ± SD. Other results are reported as mean ± SE. Data are presented for *n* = 9, unless otherwise indicated. Differences between hyperinsulinemic euglycemic clamps before, after and long term were analyzed with a Friedman test. Differences between meal tests before and after were analyzed with a Wilcoxon matched-pairs signed rank test. Differences between muscle gene expression in the current and previous study were analyzed with a Mann–Whitney test. All statistical tests were performed two sided, with the statistical significance was set at *p* < 0.05. Statistical analyses were performed using IBM SPSS version 23.0 for MacOSx (IBM, Armonk, NY, USA).

### Reporting summary

Further information on research design is available in the Nature Research Reporting Summary linked to this article.

## Supplementary information

Supplementary Information

Reporting Summary

## Data Availability

Source data are provided with this paper and are available from the corresponding author upon reasonable request.

## Source data

Source Data

## Code availability

Insights in the details of the code is available upon request.
