# Supplementary material for: Metabolic responses to mild cold acclimation in type 2 diabetes patients
Source: Nat Commun. 2021 Mar 9;12:1516. doi: 10.1038/s41467-021-21813-0 (PMC7943816; doi:10.1038/s41467-021-21813-0)
Supplement: Supplementary file 1 — Supplementary Information [file 41467_2021_21813_MOESM1_ESM.docx]

**Supplementary figures and tables**

**Supplementary Table 1. Participant characteristics regarding diabetes**

| Participant | Diabetes duration (years) | Diabetes medication |
| --- | --- | --- |
| 1 | 15 | Metformin 850mg, 3x/day  Gliclazide 80mg, 2x/day |
| 2 | 14 | Metformin 500mg, 2x/day |
| 3 | 15 | Metformin 1000mg, 2x/day  Gliclazide 30mg, 2x/day |
| 4 | 6 | Metformin 1000mg, 3x/day |
| 5 | 8 | Metformin 850mg 1x/day  Glimepiride 2mg, 1x/day  Liraglutide 6mg, 18eh, 1x/day |
| 6 | 5 | Metformin 850mg, 2x/day |
| 7 | 9 | Metformin 500mg, 2x/day |
| 8 | 7 | Metformin 500mg, 3x/day  Gliclazide 30mg, 1x/day |
| 9 | 1 | Gliclazide 80mg, 1x/day |

**Supplementary Table 2. Nutritional information of meal test shake**

|  | Content | Energy % |
| --- | --- | --- |
| Energy | 755 kcal | 100 |
| Fat | 50.8 gr | 60.6 |
| Saturated fat | 26.1 gr | 31.1 |
| Unsaturated fat | 23.8 gr | 28.4 |
| Cholesterol | 0.9 gr | 1.1 |
| Carbohydrate | 62.3 gr | 33.0 |
| Protein | 10.9 gr | 5.8 |

**Supplementary Table 3.** Plasma levels and substrate kinetics measured during the hyperinsulinemic euglycemic clamps

|  | **HE-clamp before** | **HE-clamp after** | **HE-clamp**  **long term** | **p-value** |
| --- | --- | --- | --- | --- |
| **Plasma Glucose** (mmol l^-1^) |  |  |  |  |
| Baseline | 7.77 ± 0.54 | 7.98 ± 0.45 | 7.91 ± 0.54 | 0.99 |
| Low insulin | 5.99 ± 0.25 | 5.78 ± 0.31 | 5.63 ± 0.17 | 0.21 |
| High insulin | 5.21 ± 0.12 | 5.11 ± 0.11 | 5.04 ± 0.08 | 0.69 |
| **Plasma Insulin** (mU l^-1^) |  |  |  |  |
| Baseline | 6.32 ± 1.992 | 6.99 ± 1.48 | 6.56 ± 1.71 | 0.69 |
| Low insulin | 14.09 ± 1.87 | 12.64 ± 1.56 | 12.62 ± 0.88 | 0.57 |
| High insulin | 62.50 ± 3.24^C^ | 59.34 ± 4.13 | 56.03 ± 2.68^C^ | **0.02*** |
| **Energy expenditure** (kJ min^-1^) |  |  |  |  |
| Baseline | 4.82 ± 0.25 | 4.91 ± 0.27 | 4.86 ± 0.20 | 0.79 |
| Low insulin | 4.86 ± 0.34 | 4.92 ± 0.28 | 4.75 ± 0.24 | 0.24 |
| High insulin | 4.67 ± 0.30^B^ | 4.89 ± 0.27^B,D^ | 4.67 ± 0.18^D^ | **0.02*** |
| **Carbohydrate oxidation**  (μmol kg^-1^ min^-1^) |  |  |  |  |
| Baseline | 3.76 ± 0.47^A^ | 4.97 ± 0.68^A^ | 4.68 ± 0.68 | **<0.01**** |
| Low insulin | 7.27 ± 0.79 | 7.83 ± 0.84 | 7.15 ± 0.77 | 0.79 |
| High insulin | 10.36 ± 1.15 | 11.07 ± 1.21 | 9.73 ± 1.41 | 0.30 |
| **Fatty acid oxidation**  (μmol kg^-1^ min^-1^) |  |  |  |  |
| Baseline | 3.80 ± 0.17^A^ | 3.57 ± 0.17^A,D^ | 3.71 ± 0.19^D^ | **<0.01**** |
| Low insulin | 2.81 ± 0.23 | 2.86 ± 0.24 | 2.96 ± 0.16 | 0.87 |
| High insulin | 2.14 ± 0.24 | 2.16 ± 0.21 | 2.39 ± 0.26 | 0.99 |

Abbreviations: HE-clamp, hyperinsulinemic euglycemic clamp; Ra, rate of appearance; Rd, rate of disappearance; S_i_, whole body insulin sensitivity; EGP, endogenous glucose production; NOGD, non-oxidative glucose disposal; FFA, free fatty acids.

Data are expressed as mean ± SE. n=9. Data was analyzed with a two-sided Friedman test. **p<0.01, *p<0.05, ^A^significant difference (p<0.01) between Clamp pre and Clamp post, ^B^significant difference (p<0.05) between Clamp pre and Clamp post, ^C^significant difference (p<0.05) between Clamp pre and Clamp post, ^D^trend in difference (p<0.10) between Clamp post and Clamp longterm.

**Supplementary Table 4.** Plasma levels and substrate kinetics measured during the meal tests

|  | **Meal test**  **before** | **Meal test**  **after** | **p-value** | |
| --- | --- | --- | --- | --- |
| **Plasma Glucose^1^** |  |  |  |  |
| AUC | 4433 ± 236 | 4285 ± 170 | 0.43 |  |
| iAUC | 860 ± 171 | 747 ± 159 | 0.25 |  |
| **Plasma Insulin^1^** |  |  |  |  |
| AUC | 15826 ± 1550 | 15640 ± 1458 | 0.65 |  |
| iAUC | 9457 ± 1097 | 9060 ± 1119 | 0.36 |  |
| **Plasma Triglycerides^1^** |  |  |  |  |
| AUC | 1339 ± 109 | 1411 ± 123 | 0.50 |  |
| iAUC | 655 ± 89 | 664 ± 67 | 0.36 |  |
| **Plasma Free fatty acids^1^** |  |  |  |  |
| AUC | 214852 ± 9104 | 202701 ± 10011 | 0.16 |  |
| iAUC | 13182 ± 6077 | 23031 ± 7650 | 0.20 |  |
| AUC first meal | 94228 ± 5115 | 86014 ± 4545 | **0.04*** |  |
| AUC second meal | 120624 ± 5448 | 106486 ± 11976 | 0.30 |  |
| **Energy expenditure^2^** |  |  |  |  |
| AUC | 2620 ± 145 | 2752 ± 168 | **0.03*** |  |
| iAUC | 316 ± 37 | 355 ± 22 | 0.69 |  |
| **Carbohydrate oxidation^2^** |  |  |  |  |
| AUC | 3328 ± 371 | 3866 ± 550 | 0.44 |  |
| iAUC | 726 ± 152 | 998 ± 103 | 0.22 |  |
| **Fatty acid oxidation^2^** |  |  |  |  |
| AUC | 1688 ± 92 | 1667 ± 78 | >0.99 |  |
| iAUC | 611 ± 159 | 526 ± 63 | 0.94 |  |

Abbreviations: AUC, area under the curve; iAUC, incremental area under the curve.

Data are expressed as mean ± SE, ^1^n=9 and ^2^n=7. Data was analyzed with a two-sided Mann-Whitney test. *p<0.05

**Supplementary Table 5.** Primer sequences

| **Gene** | **TaqMan AssayID** |
| --- | --- |
| *CHRNA1* | Hs00909664 |
| *CCDC39* | Hs00977326 |
| *MYH8* | Hs00267293 |
| *CCDC80* | Hs00277341 |
| *CHRND* | Hs00897937 |
| *ACTC1* | Hs01109515 |
| *COL1A1* | Hs00164004 |
| *COL3A1* | Hs00943809 |
| *MYH3* | Hs01074230 |
| *THBS4* | Hs00170261 |
| *CHRNG* | Hs00183228 |
| *SESN3* | Hs00914870 |
| *MYBPH* | Hs00192226 |
| *IGFN1* | Hs01567410 |

| **Gene** | **Forward Sequence (5'-3')** | **Reverse Sequence (5'-3')** |
| --- | --- | --- |
| *RPLPO* | CCATTCTATCATCAACGGGTACAA | AGCAAGTGGGAAGGTGTAATCC |

**a**

**b**

**Supplementary Figure 1. Subjective responses to cold acclimation**

**a** Self-reported thermal sensation and **b** thermal comfort measured with VAS scales at selected timepoints (from T=0 min until T=360 min) during day 3 and day 10 of the cold acclimation period, shown as AUC. Data are expressed as mean ± SE. n=9. Data was analyzed with a two-sided Wilcoxon matched-pairs signed rank test. No significant differences were observed (all p>0.05). Data
